# Quantitative diffusion and perfusion MRI in the evaluation of endometrial cancer: validation with histopathological parameters

**DOI:** 10.1259/bjr.20210054

**Published:** 2021-06-16

**Authors:** Serena Satta, Miriam Dolciami, Veronica Celli, Francesca Di Stadio, Giorgia Perniola, Innocenza Palaia, Angelina Pernazza, Carlo Della Rocca, Stefania Rizzo, Carlo Catalano, Silvia Capuani, Lucia Manganaro

**Affiliations:** 1 Department of Radiological, Oncological and Pathological Sciences, Umberto I Hospital,“Sapienza” University of Rome, Rome, Italy; 2 CNR Institute for Complex Systems (ISC), Physics Department, “Sapienza” University of Rome, Rome, Italy; 3 Department of Maternal and Child Health and Urological Sciences, Umberto I Hospital,“Sapienza” University of Rome, Rome, Italy; 4 Istituto di Imaging della Svizzera Italiana (IIMSI), Ente Ospedaliero Cantonale (EOC), Lugano, Switzerland; 5 Facoltà di Scienze Biomediche, Università della Svizzera Italiana, Lugano, Switzerland

## Abstract

**Objectives:**

To investigate the role of quantitative Magnetic Resonance Imaging (MRI) in preoperative assessment of tumour aggressiveness in patients with endometrial cancer, correlating multiple parameters obtained from diffusion and dynamic contrast-enhanced (DCE) MR sequences with conventional histopathological prognostic factors and inflammatory tumour infiltrate.

**Methods:**

Forty-four patients with biopsy-proven endometrial cancer underwent preoperative MR imaging at 3T scanner, including DCE imaging, diffusion-weighted imaging (DWI) and intravoxel incoherent motion imaging (IVIM). Images were analysed on dedicated post-processing workstations and quantitative parameters were extracted: *K_trans_*, *K_ep_*, *V_e_* and *AUC* from the DCE; *ADC* from DWI; diffusion *D*, pseudo diffusion D*, perfusion fraction *f* from IVIM and tumour *volume* from DWI. The following histopathological data were obtained after surgery: histological type, grading (G), lympho-vascular invasion (LVI), lymph node status, FIGO stage and inflammatory infiltrate.

**Results:**

*ADC* was significantly higher in endometrioid histology, G1-G2 (low grade), and stage IA. Significantly higher D* were found in endometrioid subptype, negative lymph nodes and stage IA. The absence of LVI is associated with higher *f* values. *K_trans_* and *V_e_* values were significantly higher in low grade. Higher D*, *f* and *AUC* occur with the presence of chronic inflammatory cells, D * was also able to distinguish chronic from mixed type of inflammation. Larger *volume* was significantly correlated with the presence of mixed-type inflammation, LVI, positive lymph nodes and stage ≥IB.

**Conclusions:**

Quantitative biomarkers obtained from pre-operative DWI, IVIM and DCE-MR examination are an *in vivo* representation of the physiological and microstructural characteristics of endometrial carcinoma allowing to obtain the fundamental parameters for stratification into Risk Classes.

**Advances in knowledge:**

Quantitative imaging biomarkers obtained from DWI, DCE and IVIM may improve preoperative prognostic stratification in patients with endometrial cancer leading to a more informed therapeutic choice.

## Introduction

### Background

Endometrial Carcinoma (EC) is the sixth most frequent malignancy in females worldwide and the most frequent gynecological cancer in Western countries.^1–3^ It is typical of the postmenopausal age, occurring in about 90% of cases after the age of 50, with an average age at diagnosis of about 63 years.^4^ The treatment of choice is bilateral hysterosalpingo-oophorectomy, with or without pelvic or para-aortic lymphadenectomy.^5^ Prognosis is variable and highly dependent on the intrinsic histopathological features derived from post-operative histological analysis. Nowadays, EC patients are placed in Risk Classes that help predict recurrence and give an indication for adjuvant therapy, based on histological type grading, FIGO (International Federation of Gynecology and Obstetrics) stage and the presence of lympho-vascular invasion (LVI).^6^ Recently, Tumour-Infiltrating Lymphocytes (TIL’s) has also been investigated, as it has been already demonstrated to be an important prognostic factor for many tumours.^7,8^ Some authors suggested that the presence of TIL’s in EC is a strong positive prognostic factor,^9–11^ being correlated with higher Disease-Free Survival and Overall Survival.^12^

Magnetic Resonance (MR) plays a pivotal role in the locoregional staging^13^ by providing qualitative diagnostic information to guide patient management.^14^ Recently several authors highlighted the growing contribution of MR as a source of quantitative biomarkers,^15,16^ and some correlation studies between quantitative MR data and prognostic factors have been performed using DWI, IntraVoxel Incoherent Motion (IVIM) and Dynamic Contrast-Enhancement (DCR-MR). Some of these reported results on Apparent Diffusion Coefficient (ADC) values to discriminate grading in endometrial cancer, in particular showing lower ADC values in Grade 3 EC.^17–19^ The IVIM model, able to distinguish true diffusion from microcirculation-related perfusion without the use of contrast medium,^20^ showed promising results in correlating with different risk classes in EC.^21^ DCE model with the analysis of semi-quantitative (AUC) and quantitative parameters (V_e_, K_trans_, K_ep_) has been considered to predict aggressiveness and probability of recurrence of EC.^22–25^ Finally, some studies pointed out the relationship between total tumour volume and EC prognostic factors, as larger volumes are associated with a higher degree of myometrial infiltration,^26^ Grade 3 and lymph node metastases.^27^ Studies with different quantitative imaging models used simultaneously on the same study population (DCE-MR and ADC; volume and ADC) have been reported in literature, resulting in a 'multiparametric' assessment of endometrial cancer.^28–30^

However, to our knowledge, no study has so far used ADC, IVIM, DCE-MR and Volume estimation to the same EC population. Moreover, diffusion and perfusion MRI is an indirect measure of medium microstructure and relies on inferences from models and estimation of the relevant parameters. Therefore, it is necessary to validate the information obtained from MRI diffusion and perfusion parameters through complementary investigations and histology.

### Objective

Aim of this study is to validate the correlation between quantitative data obtained from the mono-exponential (ADC) and bi-exponential diffusion models (D, D*, f), DCE-MR sequences (K_trans_, K_ep_, V_e_, AUC) and tumour Volume with EC histopathological parameters directly related to prognosis (histological type, grading, stage, LVI, lymph node status), in order to predict patients' Risk Classes.

Toward this goal, this study also aims to investigate the relationship between quantitative MR biomarkers and the presence of tumour-associated inflammatory infiltrate on histological analysis as a new biomarker in the prognosis of EC patients.

## Methods and materials

### Patients selection

This prospective observational study received Ethics Committee approval. Written informed consent from patients was obtained in all cases.

55 patients with first diagnosis of endometrial carcinoma were enrolled at the Department of Gynecology of our Institute between February 2019 and September 2020.

All subjects, after performing gynecological examination, transvaginal ultrasound and diagnostic hysteroscopy with histological confirmation of endometrial carcinoma, underwent MR examination at our Radiology Department for loco-regional staging of disease.

Inclusion criteria were as follows: (a) age ≥18 years, (b) female sex, (c) written informed consent, (d) biopsy-proven endometrial carcinoma, (e) surgical indication for bilateral hysterosalpingo-oophorectomy, and (f) absence of previous neoadjuvant chemo- or radiotherapy treatment.

Eleven patients were excluded during the course of the study: of these four were diagnosed with endometrial atypical hyperplasia, other four were sent to neo-adjuvant therapy for disease spread beyond the uterus (*e.g.* in case of parameter infiltration or peritoneal carcinosis), the last three decided to reject the study to perform surgery and histological analysis in other institutes.

A total of 44 patients formed our study group. Patients characteristics were summarized in Table 1.

**Table 1.** Patients characteristics

Characteristic	Number	Percentage
Total number of patients	44	
Age (years)		
Range	51–87	
Mean age ± SD	72 ± 10	
Histology		
Endometrioid	32	72,7%
Non-Endometrioid	12	27,3%
Grading		
G1-G2	28	63,6%
G3	16	36,4%
FIGO stage		
IA	16	36,4%
IB-II	14	31,8%
IB	12	27,3%
II	2	4,5%
III-IV	14	31,8%
Lymph nodes		
Negative for tumour cells	34	77,3%
Positive for tumour cells	10	22,7%
LVI		
Absent	24	54,5%
Present	20	45,5%
Inflammatory infiltrate		
Absent	6	13, 6%
Subtyping:		
Acute	0	0%
Chronic	28	63,7%
Mixed	10	22,7%
Quantification		
Mild	18	40,9%
Moderate/intense	20	45,5%
Location		
Intratumoural	16	36,4%
Peri-tumoural	12	27,3%
Intra- and Peri-tumoural	10	22,7%

### MR protocol

All examinations were performed at our Radiology Department using a 3.0 T scanner (GE Discovery MR 750, GE Healthcare, Milwaukee, WI, USA) with an eight-channel phased-array coil positioned on lower abdomen.

Patients were asked for medium bladder filling. Supine position with the feet-first mode was used. The MR protocol included: T2 Fast-Spin Echo (FSE) Weighted Imaging (WI) on sagittal, axial and coronal plane; T2 FSE WI oriented on the short axis (para-axial plane) and long axis (para-coronal plane) of the endometrial cavity; Field of View Optimized and Constrained Undistorted Single Shot (FOCUS) DWI on para-axial plane with b-values of 0–500–1000 s/mm2 to obtain ADC maps; Spin echo (SE) Echo-Planar Imaging (EPI) DWI (IVIM) (Intravoxel Incoherent Motion) on axial plane with 8 b values (0, 30, 50, 150, 500, 800, 1000, 1500 s/mm^2^); T1 Dynamic Contrast Enhancement (DCE) images on para-axial plane to obtain perfusion maps; images were acquired using a three-dimensional (3D) spoiled gradient echo pulse (LAVA) sequence with a temporal resolution of 7 s. Sequential images were obtained from 7 s before i.v. injection of contrast agent (Claricyclic, GE Healthcare; 0.2 ml gadolinium per kilogram of body weight, 2 ml s^−1^ injection speed) to 2.21 min after.

Table 2 shows the parameters of MR scanning. Before MR examination, 20 mg of joscine N-butylbromide (Buscopan; Boehringer-Ingelheim, Ingelheim, Germany) was injected intravenously to suppress bowel peristalsis.

**Table 2.** MR scanning parameters

	TR/TE (ms)	FOV (mm)	NEX	Matrix Size	Slice Thickness (mm)	Intersection gap (mm)	B-values (s/mm2)	FA (°)	Temporal resolution (s)
Axial Sagittal and Coronal FSE T2WI	3411/121	320 × 320	2	320 × 224	4	1	/	/	/
Para-axial, Para-coronal FSE T2WI	5089/127	240 × 240	2	320 × 224	4	1	/	/	/
Para-axial FOCUS DWI	2000/57	240 × 240	2	160 × 80	3,5	0	0-500-1000	/	/
Axial SE-EPI DWI (IVIM)	2000/77	300 × 300	2	160 × 192	6	1	0, 30, 50, 150, 500, 800, 1000, 1500	/	/
3D-DCE T1WI	5/2	310 × 310	1	288 × 160	3	0	/	25	7

FA, Flip angle; FOV, Field of view; NEX, number of excitations; TE, Echo time; TR, Repetition time.

## Imaging analysis

### ADC

Two radiologists with 25 (L.M.) and 4 (S.S.) years of experience in female pelvic imaging, respectively, analysed the ADC maps on a post-processing workstation (AW VolumeShare 7, GE Healthcare, Milwaukee, WI, USA).

**Figure 1.**
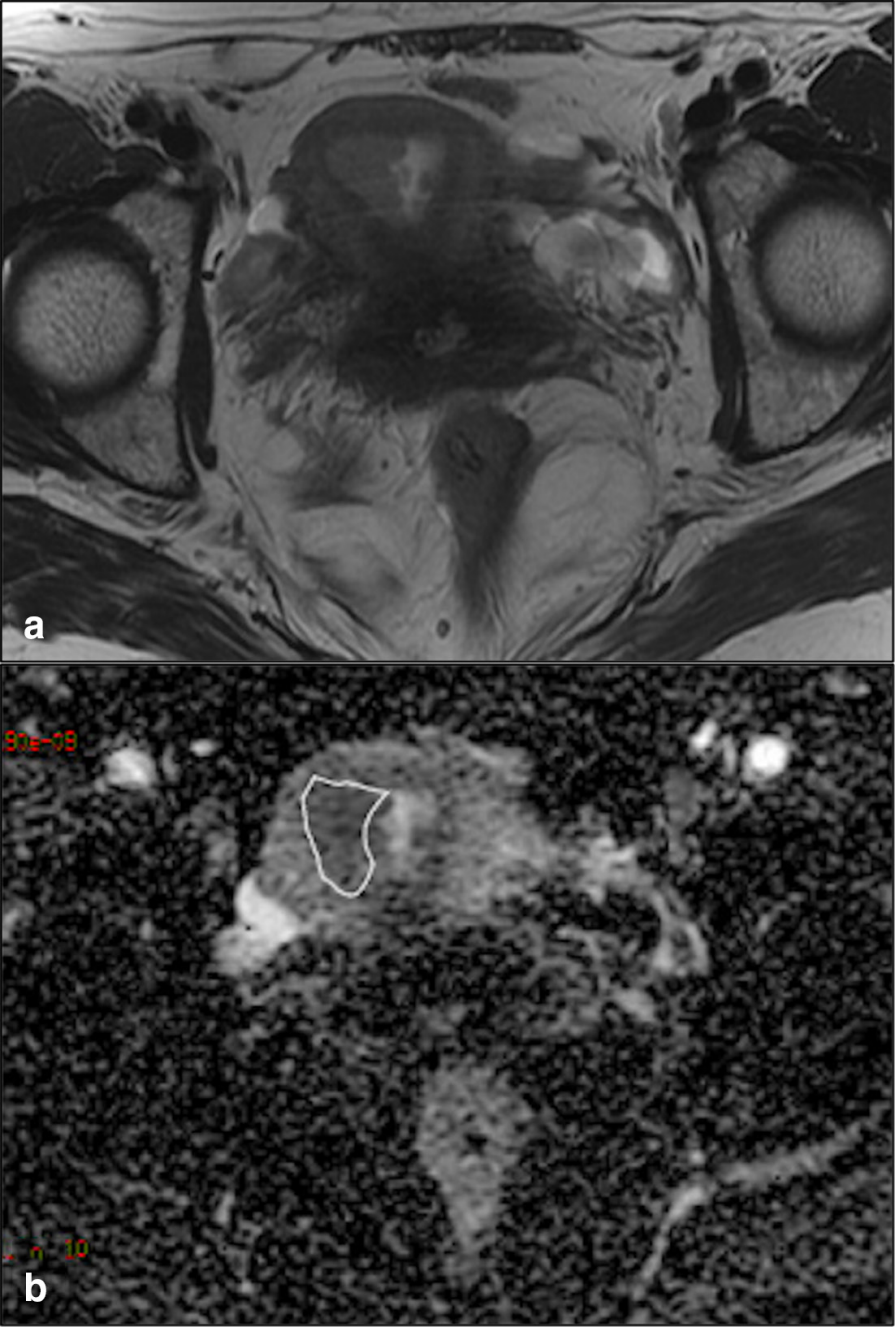
ADC maps. (**a**) Para-axial T2-WI shows tumour tissue on the right lateral side-of the endometrial cavity. (**b**) 2D-ROI (white outline) drawn at the corresponding site of the lesion on the para-axial ADC map.

Using para-axial T2-WI as a morphological reference, both radiologists independently drew three two-dimensional (2D) regions of interest on the ADC maps for each patient. ROIs were drawn in the slices containing solid tumour tissue with larger cross-sectional diameter, avoiding areas of intratumour necrosis or adjacent healthy tissue (Figure 1). Mean tumour ADC value ± standard deviation (SD) from the three ROIs was then calculated for each operator; the final ADC value was obtained from the mean ( ± SD) of the values obtained by the two operators.

#### DWI-IVIM

In the pre-processing phase, DWI images obtained at different b-values were converted from DICOM format to NIFTI (Neuroimaging Informatics Technology Initiative) format to be analysed using FSL software. Voxels corresponding to the tumour were selected for each patient. Lesion contouring was performed by a Physicist under the supervision of an experienced Radiologist (L.M., 25 years of experience in imaging of the female pelvis), using axial T2-WI as reference to avoid sampling errors such as inclusion of healthy endometrium or non-infiltrated myometrium.

Subsequently, the tumour “mask” was transferred to Matlab software (R2016a; MathWorks, Natick, MA), with cancellation of all external pixels, obtaining a VOI (volume of interest) corresponding to the pathological tissue only.

Diffusion coefficient (D), pseudodiffusion (D*) and perfusion fraction (f) values were obtained by fitting the bi-exponential IVIM function:

(1) S(b)/S(0)=(1−f)e−bD+fe−bD∗

to data obtained at different b-values. In Eq. 1, S(b) and S(0) indicate the VOI signal at b and *b* = 0, respectively; b indicates the b-values.

D quantifies the true diffusion of water molecules in the extracellular space and it is affected by tissue microstructure, D* quantifies the collective motion of blood water molecules in the capillary network, flowing from one capillary segment to the next, and f quantifies the perfusion fraction.

### Volume

Tumour volume calculation obtained on *b* = 1000 s/mm2 DWI by using the “K-means” clustering algorithm of Matlab software (R2016a; MathWorks, Natick, MA) and employing the same “masks” previously described. From the sum of the volumes of individual voxels on multiple slices, the overall tumour volume was obtained. This calculation was performed considering also the volumetric estimation of the tumour areas located in the space not sampled by the MRI sequence (slice GAP), with calculation of the possible sampling error. Assuming an ellipsoidal shape of the tumour, the number of voxels in the unsampled area is estimated to be equal to the average of the number of voxels present in the immediately upper and lower slices.

### DCE-MR

Analyses on DCE-MR images were performed separately by two radiologists (L.M.; M.D.; respectively, 25 years and 3 years of experience on female pelvis imaging) using dedicated post-processing software (Olea Sphere 3.0, Olea Medical Solutions, La Ciotat, France), based on the Tofts model. After manual selection of the arterial input function (AIF) on T1-DCE para-axial sequences, perfusion maps were obtained. Using the T2W images as morphological reference, the two radiologists manually drew on perfusion maps a two-dimensional ROI on the slice where the tumour had the largest transverse diameter, always trying to avoid adjacent healthy tissue or any areas of intratumoural necrosis.

**Figure 2.**
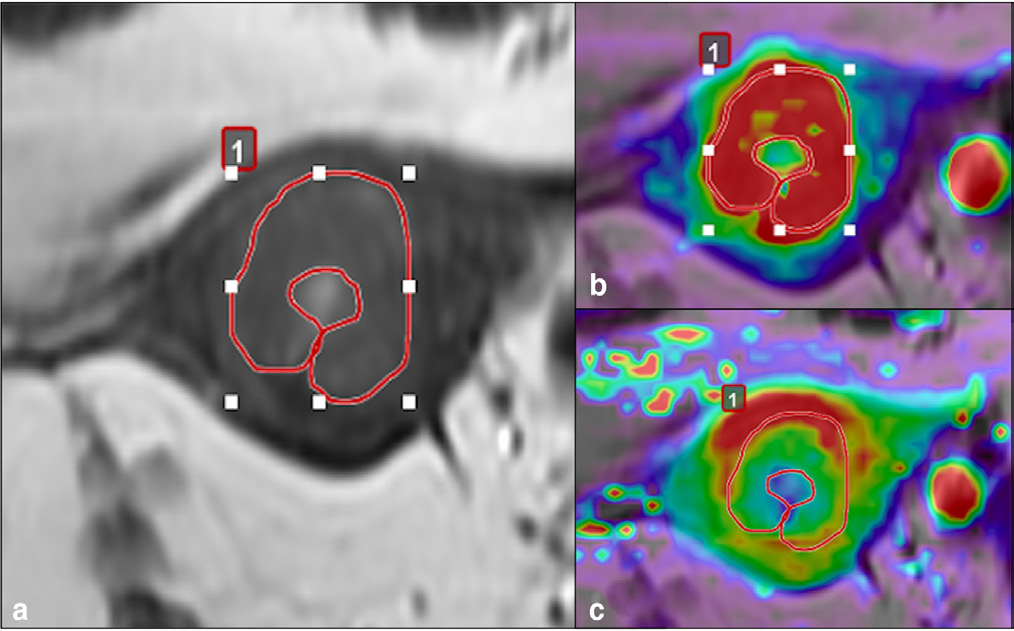
DCE-MR perfusion analysis. (**a**) T2-WI showing a 2D-ROI drawn on a large endometrial tumour, the same ROI was then applied to K_trans_ (**b**) and K_ep_(c) perfusion maps.

K_trans_, K_ep_, V_e_ and AUC values of the tumour tissue were thus extracted (Figure 2).

The final value associated with each parameter was calculated from the mean ( ± SD) of the values obtained separately by the two operators.

### Surgical treatment and histological analysis

Within 1 month from MR examination, all patients underwent surgical treatment consisting of bilateral hysterosalpingo-oophorectomy; pelvic or para-aortic lymphadenectomy was reserved only for patients with suspicion of lymph node metastases on preoperative imaging. Peritoneal washing was also performed and revealed absence of malignant cells in all cases.

Gross specimens were analysed and for each tumour sample, haematoxylin-eosin-stained slides were reviewed by two pathologists. A comprehensive histological assessment of each lesion was performed which included: histological type, grading (nuclear and architectural), myometrial infiltration (<or ≥50%), presence or absence of LVI, inflammatory infiltrate, lymph node status and FIGO stage.

In the assessment of the inflammatory infiltrate, the following were also distinguished: (a) the composition, subdividing them into acute type (neutrophilic and eosinophilic granulocytes), chronic type (B and T cells) or mixed type; (b) the disposition (intratumoural, peri-tumoural or both); (c) the quantification (mild and moderate/intense) (Table 1).

**Figure 3.**
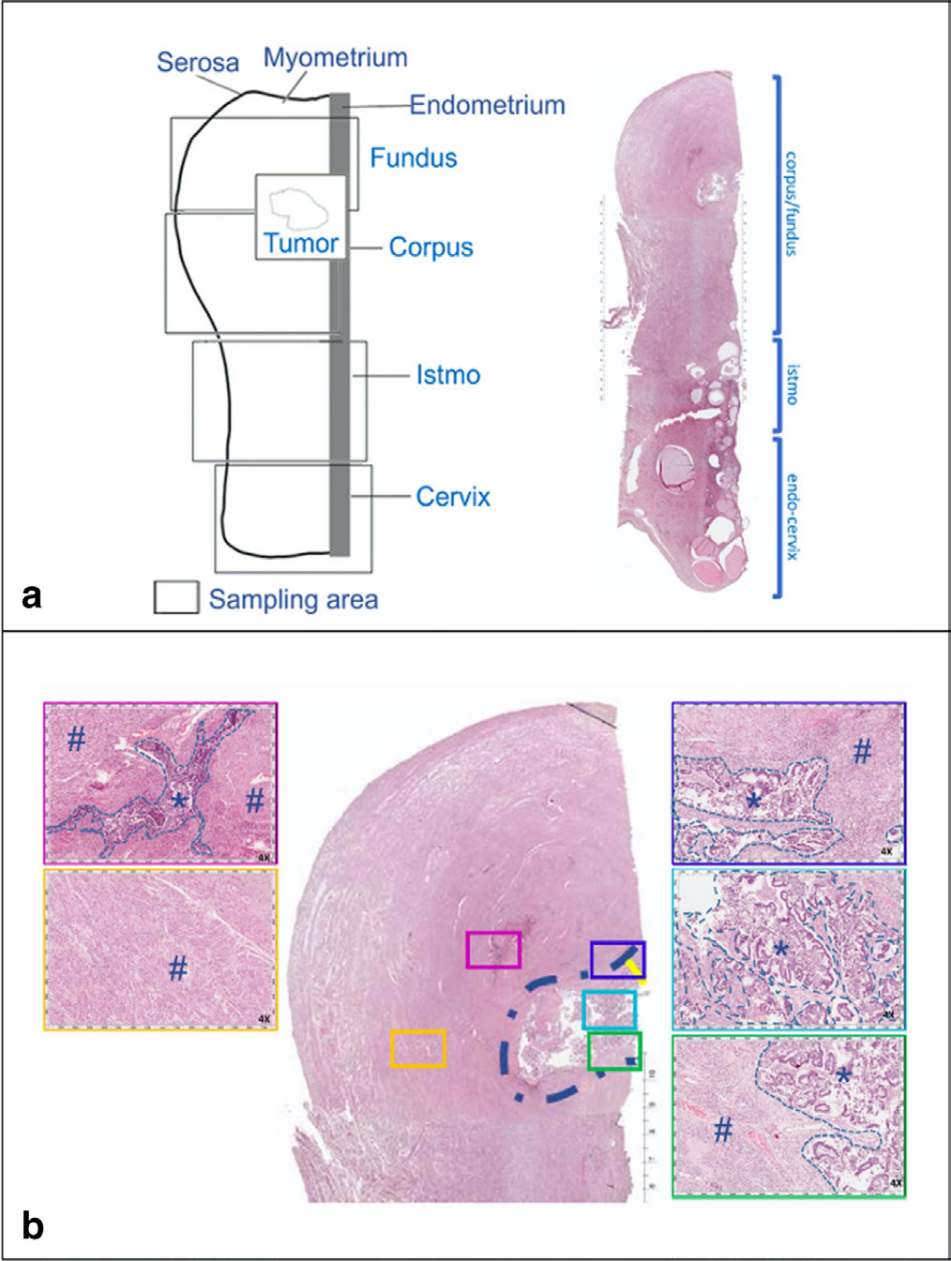
Uterine gross specimen. (**a**) Model of reconstruction of gross specimen with identification of sampling areas, later transformed into histological sections stained with H&E. (**b**) Section of the uterine fundus, the neoplastic lesion is outlined by the dotted line while in the boxes the normal myometrium (#) is differentiated from the tumour tissue (*).

Finally, a unique image of the specimen was obtained through the assembling of virtual slide derived from single histological section of tissue pieces (Figure 3). The sections were scanned through a bright-light microscope (magnification available: 4X, 10X and 20X) provided to the Aperio Digital Pathology System (slide scanner-Leica Biosystems). After recompositing in one picture, the 2D images resulted superimposable to the gross specimen and thus comparable to radiologic findings.

### Statistical analysis

Differences between mean values of quantitative MR parameters quantified in case of qualitative two- or more- modality variables (endometrioid *vs* non-endometrioid histological type, Grade 1/2 *vs* 3, presence or absence of LVI, negative/positive lymph nodes, FIGO stage, inflammatory infiltrate characteristics, extent of inflammatory infiltrate and location) were assessed with *Analysis of Variance* (*ANOVA*) *test* with *Brown and Forsythe test for the analysis of variance. Tuckey* and *Tamhane post-hoc tests* to correct for multiple comparison.

All statistical analyses were performed using SPSS 25.0 software (IBM, Armonk, NY, USA), considering a *p*-value less than or equal to 0.05 as an indicator of statistical significance.

## Results

### ADC

A statistically significant difference (*p* = 0.03) was found between ADC values quantified in case of Endometrioid (E) and Non-Endometrioid (NE) histological type. In particular, lower ADC values were associated with NE histological type, with worse prognosis (E: ADC mean value 702.54 ± 158.69; NE: ADC mean value 595.22 ± 90.75). Moreover, ADC values were statistically different (*p* = 0.02) when obtained in Grade 1–2 and Grade 3 tumour (G1-2: mean ADC value 706.80 ± 170.25; G3: mean ADC value 614.59 ± 82.70), with lower ADC values in cases with higher tumour grade.

Finally, the mean ADC value showed a decreasing trend with increasing FIGO stage of disease. A significant difference (*p* = 0.05) was found when comparing ADC values of Stage IA and Stage III-IV tumours.

No significant differences were found between ADC values quantified in tumour of subjects with LVI, positive lymph node or tumour inflammatory infiltrate.

### IVIM parameters

D* (coefficient of pseudo diffusion) was significantly different when comparing E and NE histological type (*E*: 12.67 ± 10.38 vs *NE*: 7.10 ± 2.75; *p* < 0.01), with higher values in the former. Mean D* value was also able to significantly discriminate patients with positive lymph nodes from patients with negative lymph nodes (*+ve*: 4.82 ± 0.58 vs *-ve*: 13.01 ± 9.81; *p* < 0.01); and stage IA from advanced stages III-IV (*IA*: 15.37 ± 10.48 vs *III-IV*: 5.92 ± 1.90; *p* < 0.01), in both cases with higher values associated with better prognosis.

Moreover, the perfusion fraction f showed a statistically significant correlation with the lympho-vascular invasion variable (LVI absent: mean 0.24 ± 0.12; LVI present: 0.17 ± 0.05), with higher mean values in the group with LVI absent (*p* < 0.01).

Both D* and f also showed statistically significant differences related to tumour inflammatory infiltrate: both parameters are in fact be able to discriminate tumours with a chronic-type infiltrate from tumours without inflammatory infiltrate, with significantly higher values in the first group (*p* = 0.03 and<0.01 respectively).

In the comparison between the group with chronic-type infiltrate and the mixed-type group, D* showed significantly higher values in the chronic-type group (*p* < 0.04). Moreover, a significant linear correlation was found between D* and tumour volumes (*r* = −0.483, *p* = 0.04).

No significant differences were found for D (true diffusion).

### Volume

Tumour volume demonstrated a statistically significant correlation in the assessment of LVI (LVI absent 16.65 cm^3^ ± 15.11; LVI present: 25.09 cm^3^ ± 11.99) and lymph node status (-ve lymph nodes: 18.23 cm^3^ ± 14.78; +ve lymph nodes: 28.16 cm3 ± 9.41), with higher volumes significantly associated with the two worst prognosis conditions (*p* = 0.05).

In addition, tumour volume demonstrated statistically significant differences in stage IA *vs* both IB/II (*p* < 0.01) and III/IV (*p* < 0.01).

Concerning tumour inflammatory infiltrate, larger tumour volumes are significantly associated with the presence of a mixed type infiltrate, as opposed to the chronic type (*p* = 0.04).

### DCE-MR

K_trans_ and V_e_ showed a statistically significant correlation with tumour grading (*p* = 0.01 and <0.01, respectively), with higher values associated with more differentiated G1-G2 tumours (K_trans_: 0.55 ± 0.31 mL/min/100 ml; V_e_: 0.31 ± 0.13 ml) and lower values in less differentiated G3 tumours (K_trans_: 0.32 ± 0.25 mL/min/100 ml; V_e_: 0.18 ± 0.09 ml).

We found higher K_trans_ values in patients with negative lymph nodes, but this difference was not significant (*p* > 0.05).

No significant correlations were found between quantitative perfusion parameters and histological type, LVI, FIGO stage.

No quantitative parameter of DCE-MR (K_trans_, K_ep_, V_e_) demonstrated a significant correlation with tumour inflammatory infiltrate. Only the semi-quantitative parameter AUC (area under the signal intensity/time curve) showed significantly higher values in presence of chronic-type infiltrate, compared with tumours without inflammatory infiltrate (*p* < 0.01).

No significant correlation was found between DCE and IVIM f and D* parameters. However, a positive trend was found between f and Ktrans.

All results are summarized in Table 3.

**Table 3.** Results

	ADC (× 10^−6^ mm^2^/s)	IVIM			Volume (cm^3^)	DCE			
		D* (× 10^−9^ mm^2^/s)	f	D (× 10^−9^ mm^2^/s)		k_trans_(min ^-1^)	K_ep_ (min ^-1^)	V_e_	AUC (× 10^3^)
Histological type									
E	702.54 ( ± 158.69)	12.67 ( ± 10.38)	0.20 ( ± 0.98)	0.70 ( ± 0.16)	17.49 ( ± 11.49)	0.52 ( ± 0.31)	1.66 ( ± 1.04)	0.28 ( ± 0.15)	42.44 ( ± 30.70)
NE	595.22 ( ± 90.75)	7.10 ( ± 2.75)	0.24 ( ± 0.10)	0.72 ( ± 0.14)	28.49 ( ± 18.13)	0.33 ( ± 0.27)	1.54 ( ± 1.76)	0.23 ( ± 0.08)	30.81 ( ± 25.99)
*p* value	**=0.03**	**<0.01**	=0.43	=0.42	=0.07	=0.07	=0.17	=0.35	=0.97
Grading									
G1-G2	706.80 ( ± 170.25)	10.17 ( ± 9.13)	0.22 ( ± 0.10)	0.69 ( ± 0.17)	19.61 ( ± 11.21)	0.55 ( ± 0.31)	1.84 ( ± 0.97)	0.31 ( ± 0.13)	42.55 ( ± 31.91)
G3	614.59 ( ± 82.70)	12.85 ( ± 9.57)	0.19 ( ± 0.8)	0.72 ( ± 0.14)	22.02 ( ± 18.82)	0.32 ( ± 0.25)	1.26 ( ± 1.62)	0.18 ( ± 0.09)	33.54 ( ± 25.20)
*p* value	**=0.02**	=0.78	=0.21	=0.41	=0.65	**=0.01**	=0.15	**=0.01**	=0.34
FIGO stage									
IA	748.89 ( ± 173.17)	15.37 ( ± 10.48)	0.23 ( ± 0.12)	0.70 ( ± 0.20)	9.61 ( ± 6.40)	0.42 ( ± 0.24)	1.54 ( ± 1.19)	0.20 ( ± 0.11)	34.27 ( ± 21.58)
IB/II	637.67 ( ± 125.30)	11.60 ( ± 10.15)	0.19 ( ± 0.91)	0.75 ( ± 0.12)	28.15 ( ± 16.72)	0.38 ( ± 0.25)	1.36 ( ± 0.89)	0.23 ( ± 0.10)	40.98 ( ± 23.01)
*p* value	=0.94	=0.68	=0.50	=0.41	**<0.01**	=0.38	=0.27	=0.84	=0.63
IA	748.89 ( ± 173.17)	15.37 ( ± 10.48)	0.23 ( ± 0.12)	0.70 ( ± 0.20)	9.61 ( ± 6.40)	0.42 ( ± 0.24)	1.54 ( ± 1.19)	0.20 ( ± 0.11)	34.27 ( ± 21.57)
III/IV	622.45 ( ± 125.29)	5.92 ( ± 1.90)	0.20 ( ± 0.80)	0.67 ( ± 0.13)	25.25 ( ± 10.68)	0.60 ( ± 0.40)	1.63 ( ± 1.26)	0.37 ( ± 0.13)	43.29 ( ± 42.35)
*p* value	**=0.05**	**<0.01**	=0.50	=0.41	**<0.01**	=0.38	=0.27	=0.84	=0.63
Nodal Status									
-ve	683.18 ( ± 152.34)	13.01 ( ± 9.81)	0.20 ( ± 0.11)	0.72 ( ± 0.16)	18.23 ( ± 14.78)	0.46 ( ± 0.28)	1.70 ( ± 1.33)	0.23 ( ± 0.12)	37.37 ( ± 21.30)
+ve	639.59 ( ± 145.77)	4.82 ( ± 0.58)	0.22 ( ± 0.08)	0.67 ( ± 0.11)	28.16 ( ± 9.41)	0.49 ( ± 0.42)	1.40 ( ± 0.99)	0.37 ( ± 0.13)	45.75 ( ± 49.79)
*p* value	=0.69	**<0.01**	=0.20	=0.14	**=0.05**	=0.79	=0.50	=0.93	=0.61
LVSI									
Absent	681.73 ( ± 185.51)	12.44 ( ± 9.53)	0.24 ( ± 0.12)	0.71 ( ± 0.15)	16.65 ( ± 15.11)	0.43 ( ± 0.27)	1.48 ( ± 1.13)	0.25 ( ± 0.14)	36.38 ( ± 20.26)
Present	663.11 ( ± 96.36)	9.61 ( ± 8.94)	0.17 ( ± 0.05)	0.70 ( ± 0.16)	25.09 ( ± 11.99)	0.51 ( ± 0.35)	1.81 ( ± 1.41)	0.28 ( ± 0.13)	42.74 ( ± 38.35)
*p* value	=0.69	=0.35	**<0.01**	=0.98	**=0.05**	=0.43	=0.39	=0.44	=0.51
Inflammatory Infiltrate									
Absent	643.73 ( ± 86.47)	6.94 ( ± 2.35)	0.14 ( ± 0.03)	0.69 ( ± 0.22)	18.89 ( ± 9.55)	0.43 ( ± 0.31)	2.29 ( ± 2.27)	0.25 ( ± 0.07)	16.19 ( ± 56.54)
Chronic	661.12 ( ± 175.10)	13.31 ( ± 10.79)	0.23 ( ± 0.11)	0.70 ( ± 0.15)	18.07 ( ± 16.14)	0.38 ( ± 0.27)	1.24 ( ± 1.03)	0.24 ( ± 0.13)	36.30 ( ± 22.24)
*p* value	=0.46	**=0.03**	**<0.01**	=0.10	=0.99	=0.94	=0.68	=0.29	**<0.01**
Mixed	725.01 ( ± 88.43)	7.63 ( ± 2.46)	0.17 ( ± 0.42)	0.74 ( ± 0.12)	28.21 ( ± 7.46)	0.71 ( ± 0.32)	2.32 ( ± 0.48)	0.34 ( ± 0.16)	61.46 ( ± 42.20)
Chronic	661.12 ( ± 175.10)	13.31 ( ± 10.79)	0.23 ( ± 0.11)	0.70 ( ± 0.15)	18.07 ( ± 16.14)	0.38 ( ± 0.27)	1.24 ( ± 1.03)	0.24 ( ± 0.13)	36.30 ( ± 22.24)
*p* value	=0.46	**=0.04**	=0.69	=0.10	**=0.04**	=0.09	=0.10	=0.29	=0.27

ADC, Apparent diffusion coefficent; AUC, Area under the gadolinium concentration time curve; D, Diffusion; D*, Pseudo diffusion; DCE, Dynamic contrast-enhanced; E, Endometrioid; FIGO, International federation of gynecology and obstetrics; G, Grade; IVIM, Intravoxel incoherent motion; K_ep_, Rate constant from extravascular extracellular space to blood; K_trans_, Transfer constant from blood to extravascular extracellular space; LVSI, Lympho-vascular space invasion; NE, Non-endometrioid; V_e_, Fractional volume of extravascular extracellular space; f, Perfusion fraction; -ve, Negative;+ve, Positive.

Differences between quantitative MRI parameters in different histological groups. Statistically significant differences in light grey boxes. Results are expressed as mean value ± standard deviation in brackets.

## Discussion

Endometrial cancer is a very heterogeneous malignancy with a highly variable prognostic outcome, dependent on multiple histopathological and genetic factors.^6,31,32^

In this study, by using different quantitative MR imaging techniques (ADC, IVIM, DCE-MR and MR-Volumetry), we attempted to identify and validate some imaging biomarkers in the assessment of histopathological parameters that could provide a prognostic impact, including tumour inflammatory infiltrate.

As a first result, in agreement with previous ADC studies^17–19,29^ we observed that ADC is able to identify tumours with a worse prognosis. In particular, lower mean ADC values were observed in patients with non-endometrioid histological type, or in patients with poorly differentiated tumours (Grade 3) or in more advanced stages (FIGO stage III/IV).

In current clinical trials, ADC is the default measure of mean diffusivity of water in tissues, which assumes monoexponential DWI signal decay with increasing b-values from 0. Lower ADC values generally indicate hypercellular tissues where diffusion is restricted. However, the ADC parameter is also partially affected by perfusion. Therefore, in tissues where the perfusion contribution is not negligible, the ADC does not indicate the true diffusion value because it is also affected by the perfusion which increases ADC value.

On the other hands, IVIM, which assumes bi-exponential DWI signal decay with increasing b-values allows the quantification of the diffusion parameter D without perfusion bias and the estimation of the fraction (f) of water perfusing at rate D*.

In this regard, our results indicate that D quantifying the true random molecular motion is not a biomarker in the preoperative assessment of tumour aggressiveness in patients with. Indeed, no-association of D with histological type, grading (G), lympho-vascular invasion (LVI), lymph node status, FIGO stage and inflammatory infiltrate was found. Conversely, this work suggests a superiority of IVIM perfusion-related parameters in describing tumour behaviour. The different perfusion in the microcapillaries indicates different degrees and types of tumours and therefore a specific prognosis. IVIM perfusion-related parameters f and D* show significantly higher values in tumours with a better prognosis. In particular, higher mean values of D* were observed in patients with an endometrioid histological type or with myometrial infiltration <50% (FIGO stage IA) or without lymph node involvement. Referring to f, we observed higher mean values in tumours that did not present lympho-vascular space invasion on histological examination.

On this topic, there are heterogeneous data in the current literature. Liu et al^33^ observed an inverse pattern of f and D in differentiating endometrial cancer from normal tissue, in particular lower f and higher D in EC. They also found a D reduction in normal endometrium, but no significant differences of D between tumour and non-pathological tissue. Zhang et al^21^ evaluated Multi b-value diffusion weighted imaging for preoperative evaluation of risk stratification in early-stage endometrial cancer. They found higher f values in low-risk EC compared to non-low-risk (intermediate and high), and lower D and ADC values in non-low-risk tumours compared to the low-risk category.

The difference between our results and those of Liu et al^33^ and Zhang et al^21^ is probably due to the fact that in both of their works data with higher b values (up to 3000 s/mm^2^ and up to 2000 s/mm^2^, respectively) than those used in our work (up to 1500 s/mm^2^) were acquired. The more b increases, the more the signal is sensitive to more restricted dynamics and therefore to variations in tissues with higher cell density.

According to our results, high-risk EC showed reduced intra tumour perfusion phenomena. This may be supported by the fact that tumours with aggressive features, such as non-endometrioid subtype, Grade 3 and advanced FIGO stage, grow rapidly without adequate neoangiogenesis (*i.e.* blood support) leading to tissue hypoxia. The lack of oxygenation of cells with a high metabolism, such as neoplastic cells, leads to necrosis within the tumour, resulting in areas of hypoperfusion.^34^ In this regard we also found a significant negative correlation between D* and tumour volume. As a consequence, larger tumour volumes generated by rapidly growing tumour are characterized by hypo-perfusion. In our radiological experience, at a qualitative assessment, the most aggressive tumours show in most cases areas of intra tumoural necrosis.

Regarding perfusion parameters derived from DCE-MR, we observed significant differences in K_trans_ and V_e_ values in the various histological grades, specifically higher values of K_trans_ and V_e_ were found in Grade 1–2 tumours than in Grade 3 tumours. These results are in agreement with IVIM results, confirming once again that the most aggressive tumours are characterized by hypercellularity, reduced extracellular space and therefore hypoperfusion, resulting in reduced K_trans_ (representing the transfer from the blood to extravascular extracellular space) and V_e_ (representing the volume of extracellular space into which molecules exude from leak from the capillaries).

Even if the IVIM parameters f and D * indicate the same general physiological picture, with lower f and D* in Grade 3 tumours showing a reduced capillaries perfusion in EC, we did not find significant linear correlation between the DCE and IVIM parameters, but only a positive trend between Ktrans and f.

Ktrans describes the transport of contrast medium from blood vessels into the extravascular extracellular space depending on the balance between vascular permeability and blood flow in the tissue of interest.^35^ On the other hand, in IVIM model, f parameter indicates the percent of a voxel volume occupied by semi-randomly organized capillaries where molecules diphase at a rate quantified by D* due to perfusion.^20^ Therefore, K_trans_ and f parameters indicate not exactly the same perfusion mechanism.

Our work shows that all perfusion-dependent parameters of both DCE and IVIM have a higher value in low-grade than high-grade tumours. Also the ADC, since it depends on the diffusion but also in part on the perfusion, assumes higher values in low grade tumours.

Once again, opinions in the literature are varied on this subject as well. A study by Haldorsen et al^25^ observed that low F_b_ (tumour blood flow) and low K_trans_ were significantly associated with reduced progression/recurrence-free survival ratio. Fasmer et al^28^ found that low F_b_ and low K_ep_ (representing the transfer from the extravascular extracellular space to the blood) were significantly associated with high-risk histologic features (endometrioid Grade 3 or non-endometrioid subtype). Even although in both studies significant correlations were obtained with perfusion variables partly different from ours, the results of this study seem to be in line with our belief, confirming that hypoxia is associated with an aggressive biologic behavior of EC.

In our opinion, the differences between results of this work and some studies reported in the literature are due to: (a) differences in the MR diffusion and perfusion protocols (*e.g.* different number of b-values, different resolution), (b) selection criteria of the tumour masses, (c) the heterogeneity of the tumours analysed. Moreover, we have taken great care in validating the diffusion and perfusion parameters acquired with the microstructural and physiological characteristics of the patients through an accurate histological investigation.

In addition, concerning MR-Volumetry we observed that larger volumes were significantly represented in tumours with lympho-vascular invasion, with positive lymph nodes and in >IA stages.

According to clinical and surgical data, tumour volume is still one of the most important prognostic factors for disease progression, with an increasing frequency of negative prognostic factors as tumour volume increases, as well as a reduction in Disease-Free Survival and Overall Survival.^27^ Our results confirm this hypothesis and are perfectly concordant with previous literature results. Nougaret et al^29^ suggested that the mean tumour volume and TVR (tumour volume ratio) were significantly larger in patients with lympho-vascular invasion and in Grade 3 tumours than Grades 1 and 2. Sahin et al^26^ found that as tumour volume ratio increased, deep myometrial invasion increased.

Finally, interesting results emerged from the relationship between quantitative MR biomarkers and the presence of tumour-associated inflammatory infiltrate. Both D* and *f* showed significantly higher values in tumours with a chronic inflammatory infiltrate compared to those without inflammatory cells on histological examination. D* was also shown to be able to differentiate between chronic and mixed type infiltrate, with higher values in cases of lymphocyte prevalence. Volume was significantly reduced in tumours with chronic-type inflammatory infiltrate compared to those with a mixed type. Concerning DCE parameters, only AUC showed a significant difference in distribution between infiltrate types, with higher AUC values in tumours with chronic inflammatory infiltrate than in those without inflammatory cell.

Our results should be interpreted keeping in mind that the chronic inflammatory infiltrate is predominantly represented by T and B lymphocytes, whereas the acute inflammatory infiltrate is characterized by neutrophilic and eosinophilic granulocytes.

Several studies have already investigated the role of lymphocytes in EC, showing that their presence is associated with a better clinical outcome^36^ while the absence would be a sign of poor prognosis.^37^ Furthermore, it has been reported for several tumours that the presence of neutrophils would be a negative prognostic factor, with an increased risk of recurrence.^38–40^

Our results seem to confirm both hypotheses, in fact the presence of chronic type infiltrate is associated in our work with higher values in both IVIM perfusion parameters (D* and f) and DCE (AUC), which are related to EC with a better prognosis. At the same time, mixed-type inflammatory infiltrate (also containing neutrophilic granulocytes) was found to be associated with lower D* values and higher volumes, both parameters correlated with worse prognosis.

There were some limitations to our study. Firstly, it was a single centre study with a relatively small number of patients. Secondly, not all histological analyses included tumour genomic characterization, which was therefore not included in our research. Moreover, in the histological evaluation of tumour inflammatory infiltrate, no immunohistochemical subtyping of the cell subpopulations was performed. Thirdly, in order to measure several parameters, we used three distinct post-processing software with different sampling methods. Specifically, IVIM and Volume parameters were calculated on all slices and voxels of the tumour, covering the entire three-dimensional volume; ADC values were obtained from the selection of three two-dimensional ROIs that included the largest areas of each lesion; DCE-MR data were obtained from two-dimensional ROIs drawn on a single slice that included the largest tumour cross-sectional diameter.

Further studies with a larger cohort of patients are needed and a single sampling method would be desirable to standardize and validate our results. Nonetheless, we strongly believe that our study, although preliminary, shows the important prognostic potential of “multiparametric” diffusion and perfusion MRI in EC.

## Conclusions

In this work, a meticulous study of association between diffusion and perfusion MRI parameters and histopathological parameters in patients with endometrial cancer was carried out.

Quantitative parameters obtained from pre-operative diffusion and perfusion MR examination may be a useful *in vivo* representation of the physiological and microstructural characteristics of endometrial carcinoma.

Based on the results of this study, the most important prognostic factors for EC could be predicted already at staging, helping in predicting tumour aggressiveness. Specifically, in our work we observed that higher *ADC*, D*, *f*, *K_trans_* and *V_e_* are associated with better prognosis EC, while larger volumes are associated with worse prognosis tumours.

Furthermore, our study suggests that diffusion and perfusion MRI parameters are also able to identify and differentiate the inflammatory tumour infiltrate. In particular, our results show that higher D* f and *AUC* are associated with the presence of TILs, a new promising and independent prognostic factor, being correlated with higher Disease-Free Survival and Overall Survival.
